# On Varicocele: A Practical Treatise

**Published:** 1891-09

**Authors:** 


*On Varicocele: A Practical Treatise.* By WILLIAM H. BENNETT, F.R.C.S. Pp. viii.—105. London: LONGMANS, GREEN & CO. 1891.

This monograph is the outcome of the careful study of about 250 cases of varicocele seen in hospital and private practice, and of a large number of dissections and *post mortem* examinations. The two most important points insisted upon by the author are: (1) That one cause only is concerned in the actual origin of the affection, viz.—congenital abnormality; and (2) That the best mode of operating for the radical cure is the method first described by the author in the *Lancet* (February 9th, 1889), in which he excises two or three inches of the plexus of veins, including with these, in the vast majority of cases, the spermatic artery, and then stitches the divided ends together.

The book is full of sound common sense and good practical suggestions, and it may be looked upon as containing the best and latest views on the subject.

